# Midwives’ knowledge, attitudes and practice about alcohol exposure and the risk of fetal alcohol spectrum disorder

**DOI:** 10.1186/s12884-014-0377-z

**Published:** 2014-11-05

**Authors:** Janet M Payne, Rochelle E Watkins, Heather M Jones, Tracy Reibel, Raewyn Mutch, Amanda Wilkins, Julie Whitlock, Carol Bower

**Affiliations:** Telethon Kids Institute, The University of Western Australia, Perth, Australia; Child and Adolescent Health Service, Department of Health Western Australia, Perth, Australia

**Keywords:** Midwives, Knowledge, Attitude, Practice, Alcohol, Pregnancy, Fetal alcohol spectrum disorder

## Abstract

**Background:**

Midwives are an influential profession and a key group in informing women about alcohol consumption in pregnancy and its consequences. There are no current quantitative Australian data on midwives’ knowledge, attitudes and practice in relation to alcohol consumption during pregnancy and Fetal Alcohol Spectrum Disorder. We aimed to reduce this knowledge gap by understanding midwives’ perceptions of their practice in addressing alcohol consumption during pregnancy.

**Methods:**

This cross-sectional study was conducted at 19 maternity sites across the seven health regions of country Western Australia. A questionnaire was designed following review of the literature and other relevant surveys. Midwifery managers of the maternity sites distributed questionnaires to all midwives working in their line of management. A total of 334 midwives were invited to participate in the research and (n = 245, 73.4%) of these were eligible.

**Results:**

The response fraction was (n = 166, 67.8%). Nearly all (n = 151, 93.2%) midwives asked pregnant women about their alcohol consumption during pregnancy and (n = 164, 99.4%) offered advice about alcohol consumption in accordance with the Australian Alcohol Guideline, which states “For women who are pregnant or planning a pregnancy, not drinking is the safest option”. Nearly two thirds (n = 104, 64.2%) of the midwives informed pregnant women about the effects of alcohol consumption in pregnancy, they did not always use the recommended AUDIT screening tool (n = 66, 47.5%) to assess alcohol consumption during pregnancy, nor conduct brief intervention when indicated (n = 107, 70.4%). Most midwives endorsed professional development about screening tools (n = 145, 93.5%), brief intervention (n = 144, 92.9%), and alcohol consumption during pregnancy and FASD (n = 144, 92.9%).

**Conclusion:**

Nearly all midwives in this study asked and advised about alcohol consumption in pregnancy and around two thirds provided information about the effects of alcohol in pregnancy. Our findings support the need for further professional development for midwives on screening and brief intervention. Policy should support midwives’ practice to screen for alcohol consumption in pregnancy and offer brief intervention when indicated.

## Background

### The effects of alcohol consumption in pregnancy

Alcohol is a teratogenic substance [1–5] and, when consumed during pregnancy, is associated with a range of adverse effects in the developing fetus, including spontaneous abortion [6–9], stillbirth [7,9–13], low birthweight [9,10], growth deficiency [7,8], intra-uterine growth restriction [8,9], and prematurity [8,9,14]. The umbrella term FASD describes disabilities [15,16] that include fetal alcohol syndrome (FAS), partial FAS (PFAS) and neurodevelopmental disorder-alcohol exposed (ND-AE) [17]. The prevalence of FASD has been reported as 2-5% in the USA [18] and in Canada it is estimated as 1/100 births [19]. FASDs are under-diagnosed and under-reported in Australia [20] where the prevalence is reported as 1.7/1,000 live births and 4.7/1,000 Indigenous live births [21]. Children with FASDs may experience developmental delay, deficits in intellectual functioning [22,23] and difficulties with learning, memory [24–28] and attention [24]; may have poor executive functioning [27,29] and deficits in social and adaptive functioning [27]. Prenatal alcohol exposure may also result in secondary disabilities [29–31], for example, limited opportunities for work and mental health problems such as depression and self-injury. Very few adults with FAS work and live independently [30–32], many have mental health problems, trouble with the law, inappropriate sexual behaviours, drug and alcohol problems, and experience incarceration [33,34]. These secondary disabilities may be prevented by early diagnosis and management of FASD.

The degree of harm to the fetus from prenatal alcohol exposure is influenced by the dose, pattern, and timing [35] of alcohol exposure and the stage of development of the fetus when exposed. Research evidence is inconclusive about the effects of low levels of alcohol consumption during pregnancy and its risk of harm to the fetus has not been determined [35–38]. Thus the *Australian Guidelines to Reduce Health Risks from Drinking Alcohol* Guideline 4 [39] (Australian Alcohol Guideline) aims to prevent alcohol consumption during pregnancy and applies an evidence based precautionary principle: “For women who are pregnant or planning a pregnancy, not drinking is the safest option”. It is important to prevent exposure of the fetus to alcohol - there is no cure for FASDs and their burden and cost to individuals, families, communities and society is great [40].

### Australian women and the risk of alcohol exposed pregnancy

Alcohol consumption in pregnancy is an important public health issue. In Australia, drinking alcohol is regarded as a normal and sociable activity [41,42] but consumption at harmful levels, frequently with the aim of becoming intoxicated, is also part of the drinking culture. Most Australian women drink alcohol once a month or more [43]. It is concerning that young Australian women have high rates of risky drinking [44–46] placing them at increased risk of unprotected sexual activity [47–49], and unplanned pregnancy [49–51]. Around half of pregnancies are unplanned [52,53] thus the fetus may be exposed to alcohol before pregnancy recognition [54,55].

Australian studies do not provide consistent results about patterns of alcohol consumption in pregnant women [56]. Some of this inconsistency may arise from methodological differences in the research and the lack of use of standardised and validated instruments for collecting and recording alcohol consumption in pregnancy. Of respondents to the 2010 National Drug Strategy Household Survey [57], 819 women reported being pregnant in the 12 months before the survey, 47.3% of these had consumed alcohol before pregnancy recognition and 19.5% had consumed alcohol following pregnancy recognition. The proportion of pregnant women in this study who abstained from alcohol consumption increased to 52.0% in 2010 from 40.0% in 2007 [46]. Of young women in the study aged 25 and under, 90% stopped consuming alcohol once they became aware of pregnancy, whereas only 50% of women aged 36 and older did so [57]. Other Australian research has reported that many women, including those with risky and binge drinking patterns continue to drink alcohol during pregnancy [58,59].

### Midwives’ knowledge, attitudes and practice

There are no published quantitative Australian data about midwives’ knowledge, attitudes and practice in relation to alcohol and pregnancy and its consequences on the fetus. Qualitative research with midwives (n = 12) in New South Wales indicated they routinely asked women at the initial antenatal visit about how much and how often they consumed alcohol [60]. These midwives were reluctant to discuss the risks of prenatal alcohol exposure to the fetus because they did not want to distress women and perceived a lack of knowledge and evidence in the area [60]. International research has found midwives to be knowledgeable about the recommended alcohol limits in pregnancy but not fully confident in their ability to advise women [61], not always offering advice [62], nor adhering to alcohol in pregnancy guidelines [63]. It was also reported [64] that midwives may be reluctant to intervene and take specific action, and may not apply their theoretical knowledge in clinical practice.

Barriers to practice about preventing alcohol consumption in pregnancy and its consequences may include lack of knowledge, the potential for significant constraints on their time; concerns about client sensitivity, stigmatisation [65,66], and alienation [60]; the need for additional training to enhance their assessment skills; lack of referral resources for adequately dealing with alcohol use problems once identified; and lack of knowledge about the amount of alcohol that is harmful in pregnancy [67] and specific risks associated with alcohol consumption in pregnancy [60]. Midwives have identified factors that may facilitate communication about alcohol consumption in pregnancy. These include increased knowledge about alcohol and pregnancy and FASD; knowledge about conversational techniques to use when addressing alcohol consumption in pregnancy and when alcohol-related symptoms are evident; increased time for interviewing women with risk behaviours and improved opportunities for their support, and access to written materials about alcohol and pregnancy to give to women; and the need for national guidelines and management decisions about addressing alcohol use in pregnancy [68].

### Midwives’ role and contribution to prevention of prenatal alcohol exposure

Women of childbearing age have acknowledged that health professionals have an important role in advising them about alcohol consumption in pregnancy and that health professionals’ information and advice are considered by pregnant women to be persuasive and influential [55]. A national survey conducted in Australia in 2006 of 1,103 women aged 18–45 years, investigated their knowledge, attitudes and practice about alcohol in pregnancy and FASD [49]. Nearly all the women agreed that information should be readily available to women about the effect that drinking alcohol may have on the fetus (99.4%) and that health professionals were the preferred source of this information. They also thought that health professionals should ask pregnant women how much and how often they drink alcohol (96.9%), advise on how many standard drinks are safe to drink during pregnancy (96.9%), and advise women who are pregnant or thinking of becoming pregnant to give up drinking alcohol (90.7%) [69]. Nearly all the surveyed women (98.2%) supported initiatives to inform women about the effects of alcohol consumption. This evidence indicates that women will trust the advice and information they receive from midwives who are well positioned to facilitate behaviour change through brief intervention [70] when indicated, and support women to abstain or reduce their alcohol consumption in pregnancy and appropriately refer women with alcohol-use disorders to specialist alcohol services [71]. Midwives play a crucial role in the prevention of prenatal alcohol exposure and their care has potential to reduce alcohol consumption in pregnancy [72] and subsequently FASD.

We need to understand midwives’ knowledge, attitudes and practice in relation to alcohol consumption in pregnancy and FASD so that valid research data are available to: inform the development and implementation of policy to prevent prenatal alcohol exposure and FASD; contribute to professional development for midwives; provide baseline information for future evaluation of changes in midwives’ knowledge, attitudes and practice; and increase midwives’ potential to offer greater support to pregnant women. In order to do this, we asked the question - what are midwives’ knowledge, attitudes and practice in relation to alcohol and pregnancy and FASD?

## Methods

The research commenced in January 2013 and was conducted at the Telethon Kids Institute, The University of Western Australia in collaboration with the Western Australian Country Health Service (WACHS). We established a Steering Group to provide leadership, governance, management and accountability for scientific conduct which comprised eight members (seven researchers and one consumer and community representative). A Midwifery Reference Group of eight members (six midwifery leaders and two clinical midwives) was also established to provide advice for the project and provide expert opinion, comment on interpretation of findings, contribute to the uptake of results for policy and practice, and advise on continuing professional development for midwives.

### Research sample

Following approval from the Executive Director of Nursing and Midwifery Services WACHS, we contacted midwifery managers of 19 maternity sites in the seven health regions of country Western Australia (WA) (Kimberley, Pilbara, Midwest, Goldfields, Wheatbelt, South West and Great Southern). A total of 334 midwives were invited to participate in the research.

### Questionnaire development

The literature about health professionals’ knowledge, attitudes and practice relating to alcohol consumption in pregnancy and FASD was reviewed with particular emphasis on research involving midwives. The questionnaire was based on the review of the literature and adapted from previous surveys conducted with health professionals in WA [67,73–76], and other surveys of health professionals [60,61,68,77–81]. During the questionnaire development we consulted with a WACHS Midwifery Advisor, the Steering Group, and the Midwifery Reference Group. A draft questionnaire was distributed for comment to the Steering Group, the Midwifery Reference Group, and six other specialist reviewers. It was assessed for omissions, clarity, and face validity, and comments were taken into account. It was pre-tested with five and piloted with 20 members of the Australian College of Midwives WA Branch Inc., and five child health researchers. Again, the draft questionnaire was modified taking into account the result of the pre-test and pilot study before the final 10-page online questionnaire was sent to midwives.

### The Alcohol and pregnancy questionnaire

The questionnaire comprised dichotomous ‘yes/no’, multiple choice and questions using a seven point scale. The questions included:

- participating midwives’ characteristics - gender, age, date of graduation from midwifery education, length of time worked as a midwife, length of time employed by WACHS, the health region of employment and the type and model of care of midwifery practice;
- asking about alcohol consumption in pregnancy;
- the use of screening tools to assess alcohol consumption during pregnancy;
- general advice given by midwives about alcohol consumption during pregnancy;
- information provided about the effects on the fetus, child and adult of alcohol consumption during pregnancy;
- brief intervention conducted for alcohol consumption during pregnancy and confidence in ability and time available to conduct these activities;
- midwives’ attitudes about alcohol consumption during pregnancy; and
- midwives’ potential to offer greater support to women.

### Data collection

Data were collected in June and July 2013. We asked midwifery managers to forward an email from the chief investigator to invite all midwives working within their line of management to participate in the research and to complete the online questionnaire. Included in the email was a link to the questionnaire, an invitation to participate in the research, an information sheet about the research, and a questionnaire that could be printed as a hard copy. The managers confirmed and informed the investigator of the number of midwives to whom they had forwarded the email. After two weeks, the midwifery managers were asked to forward another email to repeat this process. This second invitation to participate in the research thanked midwives who had previously responded and requested those who had not to complete the questionnaire. In addition, midwifery managers were mailed hard copies of the questionnaire and asked to distribute them to midwives to provide an accessible alternative to the online questionnaire. An opportunity to be included in a prize draw for a weekend away for two was offered to midwives who completed the questionnaire.

The Executive Director of Midwifery and Nursing Services sent an email to Regional Nurse Directors, Coordinators and Directors of Nursing, and midwifery managers employed in WACHS maternity sites requesting them to encourage all midwives to complete the questionnaire. Midwives who did not provide clinical care to pregnant women were only required to provide demographic information, knowledge of the effects associated and conditions caused by alcohol consumption during pregnancy, and general advice given to pregnant women about alcohol consumption during pregnancy.

### Data management and statistical analysis

The online questionnaire was administered from a secure server. Data from the hard copy questionnaires were entered into the online questionnaire format. A copy of answers requiring interpretation was maintained and checked independently by two researchers. The questionnaire data were converted into SPSS format and analysed using IBM SPSS Statistics [82]. For questions using a seven point scale, response categories 1-3 and 5–7 were aggregated to provide 3 point summaries. Descriptive statistics were produced.

Ethical approval for the study was obtained from the WACHS Board Research Ethics Committee. The ethics committee approved receipt of a completed questionnaire as evidence of informed consent to participate in the research. Confidentiality of data and midwives’ privacy was protected. Responses to the questionnaire were not named or identified and were stored electronically in de-identified format on password protected computers. All hard copy materials were kept under lock and key.

## Results

### Response to the survey

Of 334 midwives invited to participate in the study, 89 were excluded (1 invitation was sent to a registered nurse who was not a midwife, 1 midwife was acting in a different position, 2 were managers, 5 had resigned, 16 were included twice, 24 were casual employees and had not worked over the time period of the study, and 40 were on leave). Two hundred and forty five midwives were eligible to participate and 168 questionnaires were returned (54 online and 114 hard copies). A further 2 questionnaires were excluded (one partly completed online questionnaire that was also submitted as a hard copy, and another questionnaire that was completed by a registered nurse who was not a midwife). A total of 166 midwives participated in the study and the response fraction was 67.8%. However, before the commencement of analysis one more questionnaire was excluded as it was not completed sufficiently. The remaining data for a total of 165 participants were analysed.

### Characteristics of respondents

The majority (n = 162, 98.2%) of respondents were women, just over a quarter (n = 42, 25.5%) was aged 39 years or less and the majority (n = 117, 70.9%) was aged 40 years or more. Over half (n = 98, 59.4%) had graduated from midwifery education before the year 2000, over two thirds (n = 111, 67.3%) had worked as a midwife for 10 years or more, a third (n = 52, 31.5%) had been employed as a midwife with WACHS for 10 years or more. Nearly half (n = 77, 46.7%) were employed in the Southwest and Great Southern health regions and the majority (n = 131, 80.9%) worked in either collaborative hospital teams (n = 80, 49.4%) or general practitioner led and shared care (n = 51, 31.5%). Nearly all midwives (n = 162, 98.2%) provided clinical care to pregnant women and cared for 1–40 (mean 10.2, standard deviation 7.9) women per week (data not shown).

### Addressing alcohol consumption during pregnancy

Most midwives (n = 151, 93.2%) asked pregnant women about their alcohol consumption. Three quarters of the midwives were confident (n = 122, 75.3%) and had time (n = 121, 78.6%) to do so (Table 1). The majority (n = 139, 90.8%) endorsed the use of standardised forms to record alcohol consumption in pregnancy. Just under half (n = 66, 47.5%) reported using the AUDIT (Alcohol Use Disorder Identification Test) screening tool to assess alcohol consumption in pregnancy. A further 6.9% (n = 8) used the AUDIT-C (Alcohol Use Disorder Identification Test - Consumption). In addition, midwives sought more knowledge about conversational techniques to use when assessing alcohol consumption during pregnancy (n = 134, 86.5%) and professional development about screening tools for alcohol consumption (n = 145, 93.5%) (Table 2). Nearly all of the midwives (n = 164, 99.4%) provided pregnant women with advice about alcohol consumption in pregnancy that was in accordance with the Australian Alcohol Guideline [39] that was published in 2009 “… not drinking in pregnancy is the safest option”. Most midwives were confident (n = 137, 84.6%) and considered they had time (n = 125, 81.2%) to advise pregnant women about alcohol consumption in pregnancy (Table 1).

**Table 1:** **Midwives’ practice in addressing alcohol consumption during pregnancy**

	**Midwives**	
	**Number answering question**	**n (%)**
Asking about alcohol consumption in pregnancy		
Midwives asked pregnant women about alcohol consumption	162	151 (93.2)
Assessing alcohol consumption in pregnancy - use of screening tools^†^		
AUDIT (Alcohol Use Disorders Identification Test)	139	66 (47.5)
AUDIT-C (Alcohol Use Disorders Identification Test – Consumption)	116	8 (6.9)
MAST (Michigan Alcoholism Screening Test)	111	2 (1.8)
T-ACE (Tolerance, Annoyed, Cut down, Eye-opener)	112	1 (0.9)
TWEAK (Tolerance, Worry, Eye-opener, Amnesia, Cut down)	112	1 (0.9)
CAGE (Cut down, Annoyed, Guilty, Eye-opener)	113	1 (0.9)
Other	112	7 (6.3)
Advising about alcohol consumption during pregnancy - general advice^‡^		
Not drinking alcohol in pregnancy is the safest option^‡^	165	164 (99.4)
Alcohol is harmful in the first trimester	165	20 (12.1)
Try to cut down drinking alcohol	165	18 (10.9)
Don’t become intoxicated	165	15 (9.1)
Drinking alcohol occasionally is OK	165	3 (1.8)
Informing about the effects of alcohol consumption during pregnancy		
Midwives always informed pregnant women about the effects on the fetus and child of consuming alcohol during pregnancy	162	104 (64.2)
Offering brief intervention for alcohol consumption during pregnancy		
Midwives offered brief intervention when indicated	152	107 (70.4)
WACHS policies, procedures or operational directives advising assessment of alcohol consumption during pregnancy		
Midwives aware of policies, procedures or operational directives	162	99 (61.1)
Brief intervention training		
Midwives had completed brief intervention training as part of professional development within the last 2 years	154	54 (35.1)
Midwives who were confident to…		
Advise pregnant women about alcohol consumption during pregnancy	162	137 (84.6)
Explain to pregnant women the effects on the fetus and child of alcohol consumption during pregnancy	162	125 (77.2)
Assess alcohol consumption during pregnancy	162	122 (75.3)
Conduct brief intervention for alcohol consumption during pregnancy	162	97 (59.9)
Midwives who had sufficient time to…		
Advise pregnant women about alcohol consumption during pregnancy	154	125 (81.2)
Explain to pregnant women the effects on the fetus and child of alcohol consumption during pregnancy	154	123 (79.9)
Assess alcohol consumption during pregnancy	154	121 (78.6)
Conduct brief intervention for alcohol consumption during pregnancy	152	95 (62.5)

^†^More than one screening tool could be nominated.

^‡^Percentages sum to more than 100% as multiple responses were permitted.

**Table 2:** **Midwives’ potential to offer greater support to women**

	**Midwives**	
	**Number answering question**	**n (%)**
Midwives could offer greater support if they had…		
Professional development about screening tools for alcohol consumption	155	145 (93.5)
Professional development about brief intervention	155	144 (92.9)
Professional development about alcohol consumption during pregnancy and Fetal Alcohol Spectrum Disorder	155	144 (92.9)
More accessible specialist alcohol services for referring pregnant women	153	141 (92.2)
More involvement in early pregnancy care	154	143 (92.9)
Standardised forms to record health, medical and alcohol consumption history	153	139 (90.8)
More involvement in antenatal care	155	139 (89.7)
Prompts for brief intervention	155	139 (89.7)
Web-based educational resources for midwives about alcohol consumption during pregnancy and Fetal Alcohol Spectrum Disorder	154	138 (89.6)
Hard-copy educational resources for midwives about alcohol consumption during pregnancy and Fetal Alcohol Spectrum Disorder	154	136 (88.3)
Greater supply of written materials to give to pregnant women	155	136 (87.7)
More standardised resources to use when running antenatal classes or parenting education programs	153	134 (87.6)
More knowledge about conversational techniques to use when addressing alcohol consumption in pregnancy	155	134 (86.5)

Nearly two thirds (n = 104, 64.2%) of midwives provided information to pregnant women about the effects of alcohol consumption on the fetus during pregnancy (Table 1) although the majority (n = 157, 96.9%) thought that such information should be readily available to women of childbearing age, and that pregnant women expect midwives to advise them about the consequences of alcohol consumption in pregnancy (n = 139, 88.8%) (Table 3). Three quarters (n = 125, 77.2%) said they were confident and had time (n = 123, 79.9%) to do so. Just over two thirds of midwives (n = 107, 70.4%) conducted brief intervention when indicated for alcohol consumption in pregnancy. Over half (n = 97, 59.9%) were confident and had time (n = 95, 62.5%) to do so (Table 1) and 89.7% (n = 139) requested prompts and professional development about brief intervention (n = 144, 92.9%) (Table 2).

**Table 3:** **Midwives’ attitudes about alcohol consumption during pregnancy**

	**Midwives**	
	**Number answering question**	**n (%)**
Midwives’ agreement with statements about asking every pregnant woman about whether they have consumed alcohol during pregnancy…		
Will identify women who need support to stop consuming alcohol during pregnancy	160	147 (91.9)
Will enable changes in behaviour and improved health outcomes for the mother and child	160	137 (85.6)
Will make it easier to diagnose or exclude alcohol related problems in the child	160	137 (85.6)
Will uncover complex problems that are difficult for midwives to address	160	88 (55.0)
Will lead to some women feeling judged	160	85 (53.1)
Will cause anxiety and guilt among women who have consumed alcohol during pregnancy	160	84 (52.5)
Will distress or anger pregnant women	159	70 (44.0)
Will threaten my relationship with pregnant women	160	21 (13.1)
Midwives agreed that…		
Information about the effect alcohol may have on the fetus should be readily available to women of childbearing age	162	157 (96.9)
Pregnant women should completely abstain from consuming alcohol	162	148 (91.4)
Pregnant women expect midwives to advise them about the consequences of alcohol consumption in pregnancy	162	139 (88.8)
Women planning to become pregnant in the near future should abstain from consuming alcohol	162	137 (84.6)
Infrequent consumption of one standard drink of alcohol during pregnancy is not harmful to the mother or fetus	162	52 (32.1)

### Midwives’ knowledge of effects associated with and conditions caused by alcohol consumption during pregnancy

We listed 14 effects on the fetus, child and adult that are associated with alcohol consumption during pregnancy and a third of midwives (n = 57, 34.8%) knew all 14 of these. We also listed nine conditions, seven of these (FASD, FAS, birth defects, neurological impairment, Alcohol-Related Neurodevelopmental Disorder, Alcohol-Related Birth Defects and failure to thrive), were caused by alcohol consumption during pregnancy. Less than half the midwives (n = 70, 42.9%) knew all of these. Most midwives were aware that alcohol consumption during pregnancy caused FAS and FASD (n = 155, 93.9% and n = 149, 90.3% respectively) and neurological impairment (n = 120, 72.7%) (Table 4). Most midwives (n = 144, 92.9%) called for professional development about alcohol consumption during pregnancy and FASD (Table 2).

**Table 4:** **Midwives’ knowledge of conditions caused and effects associated with alcohol consumption during pregnancy**

	**Midwives n = 165 (%)**
Knowledge of conditions caused by alcohol consumption during pregnancy^†^	
Fetal Alcohol Syndrome (FAS)	155 (93.9)
Fetal Alcohol Spectrum Disorder (FASD)	149 (90.3)
Alcohol-Related Neurodevelopmental Disorder (ARND)	131 (79.4)
Alcohol-Related Birth Defects (ARBD)	128 (77.6)
Neurological impairment	120 (72.7)
Birth defects	106 (64.2)
Failure to thrive	105 (63.6)
Attention Deficit Hyperactivity Disorder (ADHD)	75 (45.5)
Autism	35 (21.2)
Not sure	5 (3.0)
Knowledge of effects on the fetus and child associated with alcohol consumption during pregnancy^†^	
Delayed development	157 (95.2)
Learning disabilities	155 (93.9)
Behavioural problems	150 (90.9)
Lowered intelligence	144 (87.3)
Preterm birth	139 (84.2)
Neonatal abstinence syndrome	137 (83.0)
Structural brain damage	127 (77.0)
Disrupted school experience	125 (75.8)
Alcohol and/or other drug dependence	116 (70.3)
Attention Deficit Hyperactivity Disorder	113 (68.5)
Long term emotional disorders	111 (67.3)
Spontaneous abortion	111 (67.3)
Seizures	108 (65.5)
Legal problems	75 (45.7)
Not sure	6 (3.6)

^†^Percentages sum to more than 100% as multiple responses were permitted.

### Midwives’ attitudes about alcohol consumption during pregnancy

Most midwives (n = 137, 84.6%) believed that women planning to become pregnant in the near future, and pregnant women (n = 148, 91.4%) should completely abstain from consuming alcohol (Table 3). They also believed that asking every pregnant woman about whether they have consumed alcohol will enable changes in behaviour and improved health outcomes for the mother and child (n = 137, 85.6%), will make it easier to diagnose or exclude alcohol related problems in the child (n = 137, 85.6%), and will identify women who need support to stop consuming alcohol during pregnancy (n = 147, 91.9%). Our results also show that only 13.1% (n = 21) of midwives thought that asking every pregnant woman about whether they have consumed alcohol during pregnancy would threaten their relationship, although around half thought that it would distress or anger pregnant women; could cause anxiety and guilt; lead to women feeling judged and uncover complex problems that are difficult for midwives to address (Table 3).

### Midwives’ potential to offer greater support for pregnant women

Most midwives (n = 131, 80.9%) were working in models of care involving collaborative hospital teams, general practitioner led and shared care (data not shown) where other providers may also be involved in early pregnancy care. They told us they could increase their support of pregnant women if they were more involved in early pregnancy care (n = 143, 92.9%) and antenatal care (n = 139, 89.7%) (Table 2). Just over half (n = 88, 54.3%) were able to list one or more general practitioners, social workers or alcohol services for referring pregnant women (data not shown) and the majority (n = 141, 92.2%) said they could provide greater support to pregnant women if specialist alcohol services were available (Table 2). The midwives thought they could offer more support to pregnant women if they had greater access to web-based (n = 138, 89.6%) and hard-copy (n = 136, 88.3%) educational resources for themselves and written materials to give to pregnant women (n = 136, 87.7%) about alcohol consumption during pregnancy and FASD (Table 2). Just under a third (n = 51, 31.5%) of midwives reported they could access hard copy resources to give to pregnant women on alcohol consumption during pregnancy and FASD, and few (n = 12, 7.4%) listed internet resources (data not shown).

## Discussion

This study, conducted with midwives in country areas of WA, describes their knowledge, attitudes and practice in relation to the prevention of alcohol consumption in pregnancy and FASD. We found that nearly all the midwives asked pregnant clients about their alcohol consumption during pregnancy and offered advice about alcohol consumption in accordance with the Australian Alcohol Guideline [39] which states “For women who are pregnant or planning a pregnancy, not drinking is the safest option”. Only two thirds of the midwives provided information to pregnant women about the effects of alcohol consumption in pregnancy which may reflect their knowledge, and they did not always use screening tools to assess alcohol consumption during pregnancy, nor conduct brief intervention when indicated. Our results suggest that the midwives strongly supported provision of professional development in these areas. Nearly all the midwives thought pregnant women expect midwives to advise them about the consequences of alcohol consumption in pregnancy. However, around half of the midwives expressed attitudes that may act as barriers which should be taken into account during professional development. Our results indicate that these midwives could offer more support to pregnant women if they had greater involvement in antenatal and early pregnancy care allowing for earlier screening and intervention, more accessible specialist alcohol services for referring pregnant women, access to written materials for pregnant women and educational resources for themselves.

This study provides the only Australian data about whether midwives advise pregnant women according to the current Australian Alcohol Guideline [39]. International studies have noted that although aware of and knowledgeable about alcohol guidelines, there may be a discrepancy with midwives’ actual practice. They did not always adhere to or inform about guidelines [62–64], were not confident in their abilities to advise women about alcohol use during pregnancy, may be unable to manage affirmative responses to alcohol consumption and may be reluctant to intervene [61]. Our results show that most midwives considered they had time and were confident to advise about alcohol consumption in pregnancy, which may reflect that the Australian Alcohol Guideline [39] is easy to convey and reduces confusion in assessment and counselling of pregnant women [60]. They concurred with a study conducted in WA in 2007 where 85.8% of health professionals said they would advise “no alcohol in pregnancy is the safest choice” but differ from a study conducted in Denmark in 2009 where 61% of midwives recommended that pregnant women abstain from alcohol during pregnancy [62].

Midwives are an important group of educators who have an essential role in the prevention of alcohol consumption in pregnancy. They see nearly all women at some stage during pregnancy and need to be able to assess the risk of alcohol consumption and provide appropriate intervention [61,64] which is effective in encouraging pregnant women who consume alcohol to stop or reduce their intake [74]. It is important that midwives do not assume pregnant women know about the risks and know not to drink alcohol during pregnancy [64,83,84]. Women are reported to reduce or stop consuming alcohol following knowledge of pregnancy [55,85], yet some Australian studies have shown that many women, including those with risky and binge drinking patterns, continue to drink alcohol during pregnancy [58,59,86]. Asking about alcohol consumption in pregnancy, as nearly all midwives in this study reported doing, is the important first step in assessing and recording the level of risk. This can then lead to advising about the effects of alcohol consumption and its effects on the fetus and child, assisting pregnant women to stop or reduce alcohol consumption, and arranging for further support, referral and follow up, and treatment where this is required [71,87]. This includes providing supportive contexts that enable women, particularly those who are most vulnerable, to be able to disclose their drinking practices and seek help where it is needed.

There is strong social and cultural acceptance of alcohol consumption in Australia. Although women may be aware that alcohol can harm the fetus, they may also believe that light or social use of alcohol intake during pregnancy is acceptable [55,80] and that the effect of alcohol consumption on fetal development is reduced after 20 weeks [80]. This may in part be attributed to the previous Australian Alcohol Guideline [88] promulgated in 2001 which stated “… the risk is highest in the earlier stages of pregnancy … .” In line with this we found that 12.1% (n = 20) of midwives’ general advice to pregnant women about alcohol consumption included “alcohol is harmful in the first trimester”. Maternal age and higher levels of pre-pregnancy intake are predictors of alcohol use in pregnancy [59,68]. Women are giving birth at a later age and many have established their alcohol consumption patterns for many years before their first pregnancy and may find it difficult to stop or reduce their alcohol consumption [68]. Of concern, 32.1% (n = 52) of midwives (and 29.0% of WA health professionals in 2007) [76] believed that infrequent consumption of one standard drink of alcohol during pregnancy is not harmful to the mother or fetus. Again, it is possible that midwives remain influenced by the previous Australian Alcohol Guideline [88] that stated “… over a week, should have less than seven standard drinks … .” The current Australian Alcohol Guideline [39] provides an explanation and clearly states that “… a ‘no-effect’ level has not been established, and limitations in the available evidence make it impossible to set a ‘safe’ or ‘no-risk’ drinking level for women to avoid harm to their unborn children, although the risk to the fetus from low-level drinking (such as one or two drinks per week) during pregnancy are likely to be low.” It is imperative that information about the current Australian Alcohol Guideline is incorporated into professional development for midwives.

Women want to know the reason why they should not drink alcohol during pregnancy and welcome more information, explanation and supporting evidence [55,69]. The majority of midwives supported this and believed that information about the effect alcohol may have on the fetus should be readily available to women of childbearing age and that pregnant women expect midwives to advise them about the consequences of alcohol consumption in pregnancy. Yet, we found that just under two thirds of midwives provided this information although three quarters of midwives said they were confident and had the time to do so. Some authors have noted that midwives’ knowledge may impact on their practice [61], that they may be reluctant to apply their knowledge in their clinical practice [64] and may be unsure and think they lack knowledge of the evidence [60] of the effects of and conditions caused by alcohol consumption in pregnancy. Midwives need to be aware of the effects and consequences of alcohol consumption during pregnancy so they can explain these to pregnant women to support their advice that “… not drinking in pregnancy is the safest option”.

Over 90% of midwives were aware that alcohol consumption during pregnancy caused FASD and FAS, whereas only 69% of midwives [and general practitioners] in Denmark [62] reported this. Over 70% of these WA midwives and 44% of midwives [and general practitioners] in Denmark [62] were aware that alcohol consumption in pregnancy was associated with structural brain damage and caused neurological impairment. More midwives, over 90%, were aware that alcohol consumption in pregnancy is associated with delayed development, learning disabilities and behavioural problems. It is important that midwives understand that these effects are probably underpinned by structural brain damage and neurological impairment.

It is likely that the midwives’ attitudes about alcohol consumption in pregnancy influenced their practice [61,89] and the majority believed that women planning to become pregnant in the near future, and pregnant women should completely abstain from consuming alcohol (similar to other WA health professionals in 2007). In addition, the majority of midwives held positive beliefs that asking every pregnant woman about whether they have consumed alcohol will enable changes in behaviour and improved health outcomes for the mother and child, will make it easier to diagnose or exclude alcohol related problems in the child, and will identify women who need support to stop consuming alcohol during pregnancy. Most midwives generally aim to avoid distressing pregnant women and work to maintain their relationship with women in their care [61] and in this study 13.1% of midwives believed that asking every pregnant woman about whether they have consumed alcohol during pregnancy would threaten their relationship and, importantly, around half believed that asking about alcohol consumption will distress or anger pregnant women. Earlier research in 2005, showed that only 9.6% of WA health professionals [67] were concerned that discussing alcohol use in pregnancy would frighten or anger pregnant women, just as 8.9% of surveyed Canadian midwives were in 2002 [78]. In addition and consistent with other research with health professionals in WA [84], some midwives expressed attitudes that may act as barriers to addressing alcohol consumption during pregnancy. These were that asking every pregnant woman about whether they have consumed alcohol during pregnancy could cause anxiety and guilt, uncover problems that are difficult for midwives to address, and lead to women feeling judged, even though Australian women surveyed in an antenatal clinic reported they do not feel judged by midwives [81]. Strategies to address such attitudes including how to manage difficult conversations and emotional responses should be incorporated in professional development for midwives [89]. These will be important to assist midwives to have complex conversations about drinking behaviours, consistent with the use of brief intervention to identify support needs and facilitate behaviour change.

Our results indicate that less than half of the midwives used a screening tool to assess alcohol consumption even though WACHS had an alcohol brief intervention policy [90] advising assessment, feedback and referral for relevant patients, and a procedure [91] for using the AUDIT screening tool [92]. This result may be attributable to information on administration of the policy which stated that “only patients who have identified they drink alcohol should be asked to complete the alcohol AUDIT questionnaire”. This instruction is likely to change as it has been recently recommended in the WA FASD Model of Care [93] that all maternity providers conduct screening for alcohol consumption using the AUDIT-C tool, and in addition, the recently introduced National Woman Held Pregnancy Record [94] requires AUDIT-C scores and brief intervention to be recorded.

The majority of midwives showed interest in further developing their practice. They called for more involvement in antenatal care and early pregnancy care, more accessible specialist alcohol services for referring pregnant women and requested prompts and professional development about brief intervention. In addition, the midwives thought they could offer more support to pregnant women if they had greater access to written materials to give to pregnant women, as well as web-based and hard-copy educational resources for themselves about alcohol consumption during pregnancy and FASD. These results are consistent with other groups of WA health professionals surveyed in 2002 who requested resources to give to clients (78.8%) and resources for themselves (83.9%) [67]. They increased their knowledge, changed their attitudes and practice, and altered the advice they gave to pregnant women about alcohol consumption in pregnancy [76] following the distribution of educational resources about alcohol and pregnancy and FASD. Likewise, midwives may also benefit from the provision of educational resources about alcohol and pregnancy and FASD.

### Limitations

We had a 67.8% response fraction in this study which is similar to the response (67.5%) achieved in the survey conducted with other health professionals in WA in 2007 [76]. We were not able to contact midwives directly to administer the questionnaire or follow-up those who had not responded, which may have influenced the response fraction. We endeavoured to increase the response fraction by requesting the Executive Director of Midwifery and Nursing Services and midwifery managers to encourage midwives to participate in the study and complete the questionnaire. We also offered midwives the choice of being entered into a draw to win a weekend away for two when they completed and returned the questionnaire.

The generalisability of these results may be limited because this research was only conducted with midwives working in WACHS due to resource limitations so the results may not be applicable to midwives working in different settings. Generalisability will also be restricted if non-responders differed from participants in the study. Similar to other surveys of health professionals [67,76], our results do not cover all aspects of midwives’ practice that may be context specific. In addition, no data were available to compare the characteristics of responders and non-responders therefore we were not able to compare this sample with midwives who did not participate in the study. Our results may also be influenced by response bias as midwives who were more knowledgeable and interested in the topic of alcohol consumption during pregnancy and FASD may have been more likely to respond. This may have led to over-estimation of midwives’ knowledge, attitudes and practice about the topic. It may also be possible that midwives felt pressured to complete the questionnaire following the high-level encouragement from the Executive Director of Midwifery and Nursing Services, increasing the potential for social desirability bias.

## Conclusion

We concluded that the midwives in this study nearly always asked pregnant women about alcohol consumption and provided advice that is consistent with the current Australian Alcohol Guideline [39], but did not always inform them about the effects of alcohol consumption in pregnancy which may reflect their knowledge. In addition, they did not always use screening tools to assess alcohol consumption during pregnancy, nor conduct brief intervention when indicated. The midwives’ capacity to ask every pregnant woman about alcohol consumption was generally supported by their attitudes although some midwives expressed attitudes that may act barriers. These midwives said they could offer more support to pregnant women if they had access to educational resources for themselves and written materials for pregnant women, greater involvement in antenatal and early pregnancy care which would allow earlier screening and intervention, more accessible specialist alcohol services and pathways for referring pregnant women. The new knowledge generated in this study about midwives’ practice in country areas of WA may be used to contribute to professional development about alcohol screening and brief intervention, provide baseline data for the evaluation for future studies and to contribute to policy to develop midwives’ capacity to offer greater support to pregnant women.

Asking about alcohol consumption in pregnancy is the important first step in assessing and recording the level of risk and addressing this important public health issue. This should lead to advising about the effects of alcohol consumption and its effects in pregnancy, assisting pregnant women to stop or reduce alcohol consumption, arranging for further support, referral and follow up, and treatment where this is required [71,87]. Policy should support midwives’ practice to screen for alcohol consumption in pregnancy and offer brief intervention when indicated. Nearly all midwives indicated they wanted to provide more support for pregnant women and were interested in developing their practice and participate in professional development about screening and brief intervention, and alcohol consumption during pregnancy and FASD.

## Abbreviations

AUDIT: Alcohol Use Disorder Identification Test
AUDIT-C: Alcohol Use Disorder Identification Test (consumption)
Australian Alcohol Guideline: *Australian Guidelines to Reduce Health Risks from Drinking Alcohol* Guideline 4
FAS: Fetal alcohol syndrome
FASD: Fetal alcohol spectrum disorder
ND-AE: Neurodevelopmental disorder-alcohol exposed
PFAS: Partial fetal alcohol syndrome
WA: Western Australia
WACHS: Western Australian Country Health Service

## References

[1] Clarren SK, Smith DW (1978). The fetal alcohol syndrome. N Engl J Med.

[2] Mattson SN, Schoenfeld AM, Riley EP (2001). Teratogenic effects of alcohol on brain and behaviour. Alcohol Res Health.

[3] Streissguth AP, O'Malley K (2000). Neuropsychiatric implications and long-term consequences of fetal alcohol spectrum disorders. Semin Clin Neuropsychiatry.

[4] O'Leary C (2002). Fetal alcohol syndrome: a literature review.

[5] Riley EP, McGee CL, Sowell ER (2004). Teratogenic effects of alcohol: a decade of brain imaging. Am J Med Genet.

[6] Maconochie N, Doyle P, Prior S, Simmons R (2007). Risk factors for first trimester miscarriage: results from a UK population-based case–control study. BJOG.

[7] Sampson PD, Streissguth AP, Bookstein FL, Little RE, Clarren SK, Dehaene P, Hanson JW, Graham JM (1997). Incidence of fetal alcohol syndrome and prevalence of alcohol-related neurodevelopmental disorder. Teratology.

[8] Holman CDJ, English DR, Bower C, Kurinczuk JJ (1996). NHMRC recommendations on abstinence from alcohol in pregnancy. Med J Aust.

[9] Elliott EJ, Bower C (2008). Alcohol and pregnancy: the pivotal role of the obstetrician. ANZJOG.

[10] Burd L, Roberts D, Olson M, Odendaal H (2007). Ethanol and the placenta: a review. J Matern Fetal Neonatal Med.

[11] Aliyu MH, Wilson RE, Zoorob R, Chakrabarty S, Alio AP, Kirby RS, Salihu HM (2008). Alcohol consumption during pregnancy and the risk of early stillbirth among singletons. Alcohol.

[12] Kesmodel U, Wisborg K, Olsen SF, Henriksen TB, Secher NJ (2002). Moderate alcohol intake during pregnancy and the risk of stillbirth and death in the first year of life. Am J Epidemiol.

[13] O'Leary C, Jacoby P, D'Antoine H, Bartu A, Bower C (2012). Heavy prenatal alcohol exposure and increased risk of stillbirth. BJOG.

[14] Sokol RJ, Janisse JJ, Louis JM, Bailey BN, Ager J, Jacobson SW, Jacobson JL (2007). Extreme prematurity: an alcohol-related birth effect. Alcohol Clin Exp Res.

[15] Chudley AE, Conry J, Cook JL, Loock C, Rosales T, LeBlanc N (2005). Fetal alcohol spectrum disorder: Canadian guidelines for diagnosis. Can Med Assoc J.

[16] Barr HM, Streissguth AP (2001). Identifying maternal self-reported alcohol use associated with fetal alcohol spectrum disorders. Alcohol Clin Exp Res.

[17] Watkins RE, Elliott EJ, Wilkins A, Mutch RC, Fitzpatrick JP, Payne JM, O'Leary CM, Jones HM, Latimer J, Hayes L, Halliday J, D’Antoine H, Miers S, Russell E, Burns L, McKenzie A, Peadon E, Carter M, Bower C (2013). Recommendations from a consensus development workshop on the diagnosis of fetal alcohol spectrum disorders in Australia. BMC Pediatr.

[18] May PA, Gossage JP, Kalberg WO, Robinson LK, Buckley D, Manning M, Hoyme HE (2009). Prevalence and epidemiologic characteristics of FASD from various research methods with an emphasis on recent in-school studies. Dev Disabil Res Rev.

[19] Chudley AE (2008). Fetal Alcohol Spectrum Disorder: Counting the invisible - mission impossible?. Arch Dis Child.

[20] Elliott E, Payne J, Morris A, Haan E, Bower C (2008). Fetal alcohol syndrome: a prospective national surveillance study. Arch Dis Child.

[21] Harris KR, Bucens IK (2003). Prevalence of fetal alcohol syndrome in the top end of the Northern Territory. J Paediatr Child Health.

[22] Mattson SN, Riley EP, Gramling L, Delis DC, Jones KL (1997). Heavy prenatal alcohol exposure with or without physical features of fetal alcohol syndrome leads to IQ deficits. J Pediatr.

[23] O'Leary C, Leonard H, Bourke J, D'Antoine H, Bartu A, Bower C (2013). Intellectual disability: population-based estimates of the proportion attributable to maternal alcohol use disorder during pregnancy. Dev Med Child Neurol.

[24] Mattson SN, Riley EP (1998). A review of the neurobehavioral deficits in children with fetal alcohol syndrome or prenatal exposure to alcohol. Alcohol Clin Exp Res.

[25] Mattson SN, Riley EP, Delis DC, Stern C, Jones KL (1996). Verbal learning and memory in children with fetal alcohol syndrome. Alcohol Clin Exp Res.

[26] Mattson SN, Riley EP (1999). Implicit and explicit memory functioning in children with heavy prenatal alcohol exposure. J Int Neuropsychol Soc.

[27] Riley EP, McGee CL (2005). Fetal alcohol spectrum disorders: an overview with emphasis on changes in brain and behavior. Exp Biol Med.

[28] Burden MJ, Jacobson SW, Jacobson JL (2005). Relation of prenatal alcohol exposure to cognitive processing speed and efficiency in childhood. Alcohol Clin Exp Res.

[29] Rasmussen C (2005). Executive functioning and working memory in fetal alcohol spectrum disorder. Alcohol Clin Exp Res.

[30] Streissguth AP, Bookstein FL, Barr HM, Sampson PD, O'Malley K, Young JK (2004). Risk factors for adverse life outcomes in fetal alcohol syndrome and fetal alcohol effects. J Dev Behav Pediatr.

[31] Spohr HL, Willms J, Steinhausen HC (2007). Fetal alcohol spectrum disorders in young adulthood. J Pediatr.

[32] Streissguth AP, Aase JM, Clarren SK, Randels SP, LaDue RA, Smith DF (1991). Fetal alcohol syndrome in adolescents and adults. JAMA.

[33] Streissguth A (1997). Fetal alcohol syndrome: a guide for families and communities.

[34] Streissguth A, Barr H, Kogan J, Bookstein F, Streissguth A, Kanter J (1997). Primary and secondary disabilities in fetal alcohol syndrome. The challenge of fetal alcohol syndrome: overcoming secondary disabilities.

[35] O'Leary CM, Bower C (2012). Guidelines for pregnancy: what's an acceptable risk, and how is the evidence (finally) shaping up?. Drug Alcohol Rev.

[36] Sokol R, Delaney-Black V, Nordstrom B (2003). Fetal alcohol spectrum disorder. JAMA.

[37] Henderson J, Gray R, Brocklehurst P (2007). Systematic review of the effects of low-moderate prenatal alcohol exposure on pregnancy outcome. BJOG.

[38] O'Leary C (2005). Fetal Alcohol Syndrome. Management guidelines: developmental disability. Volume Version 2.

[39] National Health and Medical Research Council (2009). Australian Guidelines to Reduce Health Risks from Drinking Alcohol.

[40] Lupton C, Burd L, Harwood R (2004). Cost of fetal alcohol spectrum disorders. Am J Med Genet.

[41] Ministerial Council on Drug Strategy (2006). Towards safer drinking cultures 2006–2009.

[42] Burns L, Black E, Elliott E (2009). Monograph of the Intergovernmental Committee on Drugs Working Party on Fetal Alcohol Spectrum Disorders. Fetal Alcohol Spectrum Disorders in Australia: An Update.

[43] Young A, Powers J (2005). Australian women and alcohol consumption 1996–2003.

[44] Chikritzhs T, Catalano P, Stockwell T, Donath S, Ngo H, Young D, Matthews S (2003). Australian alcohol indicators, 1990–2001.

[45] Australian Bureau of Statistics (2006). Alcohol consumption in Australia: a snapshot, 2004–05.

[46] Australian Institute of Health and Welfare (2011). 2010 National Drug Strategy Household Survey Report.

[47] Gladstone J, Nulman I, Koren G (1996). Reproductive risks of binge drinking during pregnancy. Reprod Toxicol.

[48] Walker DS, Darling Fisher CS, Sherman A, Wybrecht B, Kyndely K (2005). Fetal alcohol spectrum disorders prevention: an exploratory study of women's use of, attitudes toward, and knowledge about alcohol. J Am Acad Nurse Pract.

[49] Peadon E, Payne J, Bower C, Elliott E, Henley N, O'Leary C, D'Antoine H, Bartu A (2007). Alcohol and pregnancy: women's knowledge, attitudes and practice. J Paediatr Child Health.

[50] Allard-Hendren R (2000). Alcohol use and adolescent pregnancy. MCN Am J Matern Child Nurs.

[51] Flanigan B, McLean A, Hall C, Propp V (1990). Alcohol use as a situational influence on young women's pregnancy risk-taking behaviors. Adolescence.

[52] Colvin L, Payne J, Parsons D, Kurinczuk J, Bower C (2007). Alcohol consumption during pregnancy in non-Indigenous West Australian women. Alcohol Clin Exp Res.

[53] Floyd RL, O'Connor MJ, Sokol RJ, Bertrand J, Cordero JF (2005). Recognition and prevention of fetal alcohol syndrome. Obstet Gynecol.

[54] Floyd RL, Decoufle P, Hungerford DW (1999). Alcohol use prior to pregnancy recognition. Am J Prev Med.

[55] Raymond N, Beer C, Glazebrook C, Sayal K: **Pregnant women's attitudes towards alcohol consumption.** *BMC Public Health* 2009, **9**(175)ᅟ. doi:10.1186/1471-2458-1189-1175. 10.1186/1471-2458-9-175 PMC2701426 19500375

[56] O'Callaghan FV, O'Callaghan M, Najman JM, Williams GM, Bor W (2003). Maternal alcohol consumption during pregnancy and physical outcomes up to 5 years of age: a longitudinal study. Early Hum Dev.

[57] Callinan S, Room R (2012). Alcohol consumption during pregnancy: Results from the 2010 National Drug Strategy Household Survey.

[58] Anderson AE, Hure AJ, Forder P, Powers JR, Kay-Lambkin FJ, Loxton DJ (2014). Risky drinking patterns are being continued into pregnancy: a prospective cohort study. PLOS one.

[59] Anderson AE, Hure AJ, Forder P, Powers JR, Kay-Lambkin FJ, Loxton DJ (2013). Predictors of antenatal alcohol use among Australian women: a prospective cohort study. BJOG: An International Journal of Obstetrics & Gynaecology.

[60] Jones SC, Eval M, Telenta J, Cert G, Shorten A, Johnson K (2011). Midwives and pregnant women talk about alcohol: what advice do we give and what do they receive?. Midwifery.

[61] Gilinsky A (2009). Alcohol-related health promotion in maternity services: Factors associated with midwifery practice in NHS Tayside.

[62] Kesmodel US, Kesmodel PS (2011). Alcohol in pregnancy: attitudes, knowledge, and information practice among midwives in Denmark 2000 to 2009. Alcohol Clin Exp Res.

[63] Grol R (2001). Successes and failures in the implementation of evidence-based guidelines in clinical practice. Med Care.

[64] Goransson M, Faxelid E, Heilig M (2004). Beliefs and reality: Detection and prevention of high alcohol consumption in Swedish antenatal clinics. Acta Obstet Gynecol Scand.

[65] Diekman ST, Floyd RL, De'coufle' P, Schulkin J, Ebrahim SH, Sokol RJ (2000). A survey of obstetrician-gynecologists on their patients' alcohol use during pregnancy. Obstet Gynecol.

[66] Sharpe TT, Alexander M, Hutcherson J, Floyd RL, Brimacombe M, Levine R, Mengel M, Stuber M (2004). Report from the CDC. Physician and allied health professionals' training and fetal alcohol syndrome. J Women's Health.

[67] Payne J, Elliott E, D'Antoine H, O'Leary C, Mahony A, Haan E, Bower C (2005). Health professionals' knowledge, practice and opinions about fetal alcohol syndrome and alcohol consumption in pregnancy. Aust N Z J Public Health.

[68] Holmqvist M, Nilsen P (2010). Approaches to assessment of alcohol intake during pregnancy in Swedish maternity care - a national-based investigation into midwives' alcohol-related education, knowledge and practice. Midwifery.

[69] Peadon E, Payne J, Henley N, O'Leary C, D’Antoine H, Bartu A, Bower C, Elliott E (2011). How do women want to be informed about alcohol use in pregnancy?. Book of Abstracts 4th International Conference on Fetal Alcohol Spectrum Disorders The Power of Knowledge Integrating Research, Policy, and Promoting Practice Around the World.

[70] O'Connor MJ, Whaley SE (2007). Brief intervention for alcohol use by pregnant women. Am J Public Health.

[71] Burns L, Breen C (2013). It's time to have the conversation: Understanding the treatment needs of women who are pregnant and alcohol dependent.

[72] Roche AM, Deehan A (2002). Women's alcohol consumption: emerging patterns, problems an public health implications. Drug Alcohol Rev.

[73] Elliott E, Payne J, Haan E, Bower C (2006). Diagnosis of foetal alcohol syndrome and alcohol use in pregnancy: a survey of paediatricians' knowledge, attitudes and practice. J Paediatr Child Health.

[74] Payne JM, France KE, Henley N, D'Antoine HA, Bartu AE, O'Leary CM, Elliott EJ, Bower C, Geelhoed E (2011). RE-AIM evaluation of the alcohol and pregnancy project: educational resources to inform health professionals about prenatal alcohol exposure and fetal alcohol spectrum disorder. Eval Health Prof.

[75] Payne JM, France KE, Henley N, D'Antoine HA, Bartu AE, Mutch RC, Elliott EJ, Bower C (2011). Paediatricians' knowledge, attitudes and practice following provision of educational resources about prevention of prenatal alcohol exposure and fetal alcohol spectrum disorder. J Paediatr Child Health.

[76] Payne J, France K, Henley N, D'Antoine H, Bartu A, O'Leary C, Elliott E, Bower C (2011). Changes in health professionals' knowledge, attitudes and practice following provision of educational resources about prevention of prenatal alcohol exposure and fetal alcohol spectrum disorder. Paediatr Perinat Epidemiol.

[77] Clarke M, Tough SC, Hicks M, Clarren S (2005). Approaches of Canadian providers to the diagnosis of fetal alcohol spectrum disorders. J FAS Int.

[78] Tough SC, Clarke M, Hicks M, Clarren S (2005). Attitudes and approaches of Canadian providers to preconception counselling and the prevention of fetal alcohol spectrum disorders. J FAS Int.

[79] Herzig K, Huynh D, Gilbert P, Danley DW, Jackson R, Gerbert B (2006). Comparing prenatal providers' approaches to four different risks: alcohol, tobacco, drugs, and domestic violence. Women Health.

[80] Reibel T (2011). Prevention of alcohol and tobacco use in pregnancy: Audit of WA Department of Health antenatal services use of alcohol & tobacco brief interventions in pregnancy.

[81] Seib CA, Daglish M, Heath R, Booker C, Reid C, Fraser J (2012). Screening for alcohol and drug use in pregnancy. Midwifery.

[82] IBM SPSS Statistics 19.0.2 (2010). Version 20.

[83] Kesmodel U, Schioler Kesmodel P (2002). Drinking during pregnancy: attitudes and knowledge among pregnant Danish women, 1998. Alcohol Clin Exp Res.

[84] France K, Henley N, Payne J, D'Antoine H, Bartu A, O'Leary C, Elliott E, Bower C (2010). Health professionals addressing alcohol use with pregnant women in Western Australia: barriers and strategies for communication. Subst Use Misuse.

[85] Cameron C, Davey T, Kendall E, Wilson AM,RJ (2013). Changes in alcohol consumption in pregnant Australian women between 2007 and 2011. Med J Aust.

[86] Anderson A, Hure A, Powers J, Kay-Lambkin F, Loxton D (2012). Determinants of pregnant women's compliance with alcohol guidelines: a prospective cohort study. BMC Public Health.

[87] Fiore M, Bailey W, Cohen S, Dorfman S, Goldstein M, Gritz E, Heyman R, Jaen C, Kottke T, Lando H, Mecklenburg R, Mullen P, Nett L, Robinson L, Stitzer M, Tommasello A, Villejo L, Wewers M (2000). Treating tobacco use and dependence: clinical practice guideline.

[88] National Health and Medical Research Council (2001). Australian Alcohol Guidelines: Health Risks and Benefits.

[89] Anderson P, Kaner E, Wutzke S, Funk M, Heather N, Wensing M, Grol R, Gual A, Pas L, Group (2004). WBIS: Attitudes and managing alcohol problems in general practice: an interaction analysis based on findings from a WHO collaborative study. Alcohol Alcsm.

[90] Department of Health WA Country Health Service (2010). Alcohol and Tobacco Brief Intervention Policy.

[91] Department of Health WA Country Health Service (2010). MR202E Alcohol and Tobacco Screening Tool.

[92] Babor TF, Higgins-Biddle JC, Saunders JB, Monteiro MG (2001). AUDIT the alcohol use disorders identification test: guidelines for use in primary care.

[93] Department of Health Western Australia (2013). Western Australian across sector statewide Implementation Plan for the Fetal Alcohol Spectrum Disorder Model of Care 2013–2018.

[94] Department of Health and Ageing (2013). National Woman Held Pregnancy Record. Australian Health Ministers' Advisory Council.

